# Exploring the causal relationship of gut microbiota in nonunion: a Mendelian randomization analysis mediated by immune cell

**DOI:** 10.3389/fmicb.2024.1447877

**Published:** 2024-12-16

**Authors:** Yun-fei Yu, Hai-Feng Gong, Wei-Ju Li, Mao Wu, Gang Hu

**Affiliations:** ^1^Department of Orthopedics, Wuxi Hospital of Traditional Chinese Medicine, Wuxi, Jiangsu, China; ^2^Department of Trauma Surgery, Affiliated Hospital of Qingdao University, Qingdao, Shandong, China; ^3^Department of Orthopedics, Guangdong Provincial Second Hospital of Traditional Chinese Medicine, Guangzhou, Guangdong, China

**Keywords:** gut microbiota, nonunion, immune cell, Mendelian randomization, instrumental variable

## Abstract

**Background:**

Emerging research indicates that gut microbiota (GM) are pivotal in the regulation of immune-mediated bone diseases. Nonunion, a bone metabolic disorder, has an unclear causal relationship with GM and immune cells. This study aims to elucidate the causal relationship between GM and nonunion using Mendelian Randomization (MR) and to explore the mediating role of immune cells.

**Methods:**

Using a two-step, two-sample Mendelian randomization approach, this study explores the causal link between GM and nonunion, as well as the mediating role of immune cells in this relationship. Data were sourced from multiple cohorts and consortiums, including the MiBioGen consortium. GM data were derived from a recently published dataset of 473 gut microbiota, and nonunion data were obtained from genome-wide association studies (GWAS).

**Results:**

MR analysis identified 12 bacterial genera with protective effects against nonunion and seven bacterial genera associated with a higher risk of nonunion, including Agathobacter sp000434275, Aureimonas, Clostridium M, Lachnospirales, Megamonas funiformis, and Peptoccia. Reverse MR analysis indicated that nonunion does not influence GM. Additionally, MR analysis identified 12 immune cell types positively associated with nonunion and 14 immune cell types negatively associated with nonunion. Building on these findings, we conducted mediation MR analysis to identify 24 crucial GM and immune cell-mediated relationships affecting nonunion. Notably, Campylobacter D, Megamonas funiformis, Agathobacter sp000434275, Lachnospirales, Clostridium E sporosphaeroides, and Clostridium M significantly regulated nonunion through multiple immune cell characteristics.

**Conclusions:**

To our knowledge, our research results are the first to emphasize a causal relationship between the gut microbiome and nonunion, potentially mediated by immune cells. The correlations and mediation effects identified in our study provide valuable insights into potential therapeutic strategies targeting the gut microbiome, informing global action plans.

## Introduction

Nonunion, a complex chronic bone metabolic disorder, imposes significant burdens on individuals, families, and society, especially due to the resulting pain and physical dysfunction (Brinker et al., 2013, 2017). Typically, nonunion refers to a condition where bone healing has not occurred for over 9 months or has shown no signs of healing for 3 months (Brinker et al., 2017), with the risk ranging from 1.9% to 15% depending on the site of the fracture (Mills et al., 2017; Zura et al., 2016; Ross et al., 2018). Bone healing is a continuous and dynamic process involving the removal of old or damaged bone tissue by osteoclasts, followed by replacement with new bone tissue by osteoblasts (Inchingolo et al., 2010; Kikyo, 2024). The regulatory mechanisms of bone healing involve various factors, such as genetics, gut microbiota, and immune regulation (Wildemann et al., 2021). Although the pathological processes of different types of nonunion vary, their overall features are influenced by genetic, biological, and mechanical factors affecting bone metabolism, bone resorption-formation balance, and bone remodeling processes (Dimitriou et al., 2011). The physiologically occurring inflammatory processes during bone healing are finely tuned and disturbance, prolongation and non-resolution lead to an altered cellular composition of the early fracture callus with an altered cytokine profile (Maruyama et al., 2020; Andrzejowski and Giannoudis, 2019; Copuroglu et al., 2013). This phase is markedly influenced by both localized and systemic reactions to detrimental stimuli. Despite substantial scholarly efforts to elucidate the nature of nonunion, the mechanisms driving its onset and progression continue to be poorly understood.

In recent years, the gut microbiota has garnered widespread attention as the human body's “second genome.” It plays a crucial role in the pathogenesis of diseases by regulating metabolic, endocrine, inflammatory, and immune functions (Wang et al., 2023; Inchingolo et al., 2024; Wallimann et al., 2021). Current research indicates that the GM primarily influences bone metabolism through three mechanisms: regulating nutrient absorption, modulating the immune system, and translocating microbial products across the gut epithelial barrier (Inchingolo et al., 2024; Wallimann et al., 2021; Hernandez et al., 2016). Of particular importance is its role in immune modulation. GM regulate bone health not only by interacting with immune cells within the gut lining but also by potentially allowing activated immune cells to release cytokines into systemic circulation or migrate to bone sites, thus directly affecting the activities of osteoblasts and osteoclasts involved in bone formation and resorption, respectively (Wallimann et al., 2021; Hernandez et al., 2016; Yu et al., 2021; Li et al., 2020; Grewe et al., 2022). Exploring how gut microbiota impacts bone metabolism might enhance our understanding of nonunion pathogenesis. This approach enables more effective risk assessment of nonunion in patients with conditions such as obesity, diabetes, or inflammatory bowel disease. Additionally, alterations in the gut microbiome may serve as biomarkers of bone metabolic activity and targets for using medications or probiotics to improve bone structure in treating nonunion.

The relationship between the GM and bone metabolism is complex and influenced by factors such as diet, lifestyle, medication, and environmental exposures. Mendelian Randomization enables the exclusion of potential confounders, facilitating in-depth analysis to uncover the causal relationships between GM, immune cells, and nonunion. To our knowledge, there are no direct studies linking the gut microbiome with nonunion. Therefore, by utilizing MR to rule out potential confounders, this study effectively establishes the causal impacts of GM on nonunion and assesses the mediation by immune cells, offering substantial insights and novel perspectives for the early screening, diagnosis, and treatment strategies for nonunion.

## Materials and methods

### Study design

**Figure 1:**
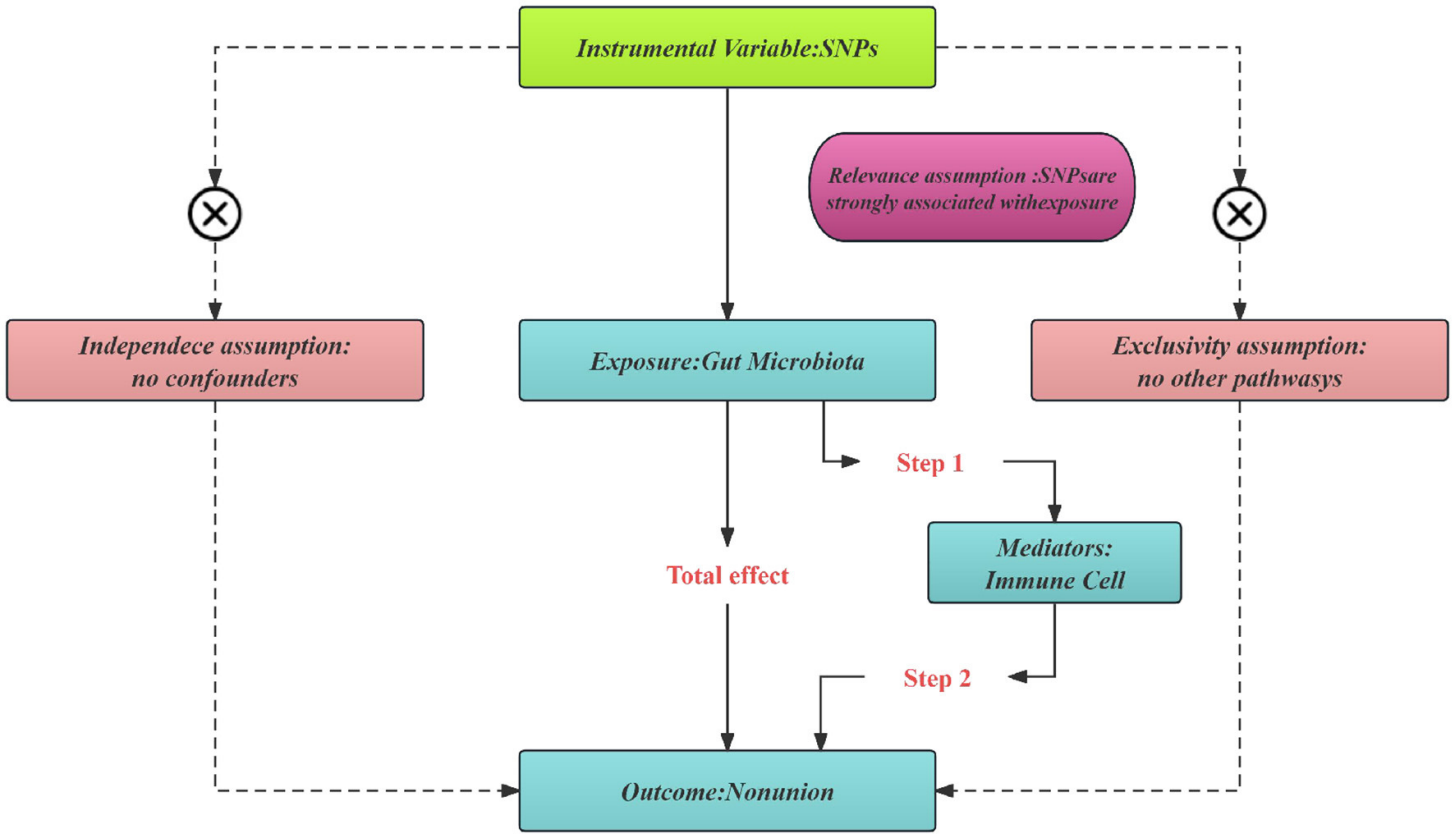
Three significant assumptions of gut microbiota on nonunion via MR.

In our study, we employed a two-sample Mendelian randomization approach (Rasooly and Peloso, 2021) to investigate the possible causal links between GM and nonunion. To deepen our understanding of the mediation by immune cells, we adopted a two-step MR strategy (Burgess et al., 2015). All studies included in the cited GWASs were approved by relevant review committees and all participants provided informed consent. The study's design and progression are illustrated in Figure 1.

### Data sources

Our research utilized data from multiple cohorts and consortiums to investigate the links between gut microbiota and nonunion. It is worth highlighting that the GWAS data for gut microbiota were obtained from the newly published 473 gut microbiote-related genome project. Complete summary statistics of GM with genome-wide significant hits are publicly available in the Genome-wide association studies (GWAS, https://gwas.mrcieu.ac.uk/). Catalog from accession GCST90032172 to GCST90032644, which includes whole-genome genotyping data from 5,959 individuals of Finnish (Qin et al., 2024). Data for 731 immune cell traits (Ebi-a-GCST0001391 to Ebi-a-GCST0002121) were from the GWAS Catalog, derived from a large-scale public GWAS (https://gwas.mrcieu.ac.uk/), which includes whole-genome genotyping data from 3,757 individuals of European Sardinia populations (Orrù et al., 2020). The summary dataset for nonunion is publicly available in the GWAS (https://gwas.mrcieu.ac.uk/). Catalog in GCST90044575, which includes whole-genome genotyping data from 456,348 individuals of European ancestry, totaling 11,831,932 SNPs (Jiang et al., 2021). All participants in the study were from Europe.

### Selection of instrumental variables

**Figure 2:**
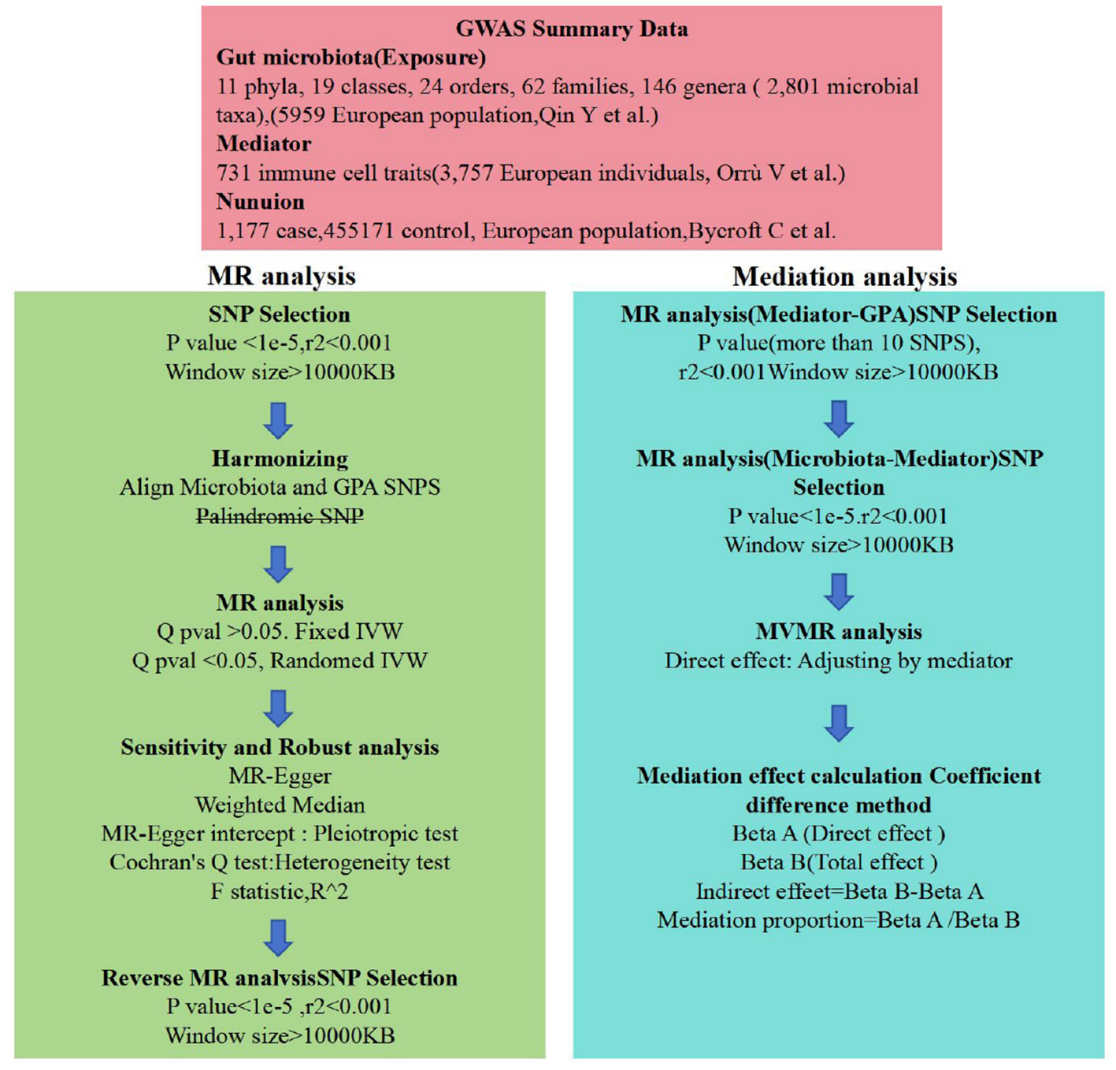
Flow chart. Outlines the methodology used to investigate the link between gut microbiota and nonuion via GWAS data. SNP selection criteria were applied before harmonization for MR analysis to determine the causal relationships and identify significant GM. Mediation analysis further quantified the potential influence of immune cell traits on the microbiota-nonunion association.

The selection of instrumental variables (IVs) for this MR analysis hinges on three core assumptions (Figure 2): (a) Instrumental variables (IVs) should be free from confounding. (b) There should be a strong link between IVs and the exposure. (c) IVs should influence the outcome exclusively through the exposure. Initially, we identified SNPs associated with each gut microbiota at a significance threshold of *P* < 1 × 10^−5^. For the mediation analysis, we adjusted the significance levels based on the count of selected SNPs being more than 10. We then employed linkage disequilibrium clumping to exclude specific SNPs that weren't desirable (*r*^2^ < 0.01, window size < 10,000 kb). Subsequently, we synchronized the datasets for exposure and outcome and eliminated palindromic SNPs with allele frequencies close to 0.5. Lastly, to quantitatively ascertain the robustness of selected SNPs as instruments, we computed the *F*-statistic for each metabolite. Generally, an *F*-statistic >10 recommends further MR analysis (Burgess et al., 2011).

### MR analysis

We conducted a bidirectional two-sample MR analysis to assess the connection between GM and nonunion. Our main analysis employed the inverse variance-weighted (IVW) meta-analysis method, a well-established technique for MR studies (Verbanck et al., 2018). To enhance the reliability of our findings, we also performed additional analyses using the MR Egger, Weighted median, Simple mode and Weighted mode (Yuan et al., 2022; Zheng et al., 2020). We evaluated the potential influence of directional pleiotropy by examining the intercept value in the MR-Egger regression (Xu et al., 2019). We gauged heterogeneity using Cochran's *Q* test (Yuan et al., 2022). Conducting single-SNP and leave-one-out analyses to evaluate the likelihood that observed associations are driven by individual SNPs. The indirect effect of GM on nonuion via potential mediator was evaluated with the “product of coefficients” method.

### Statistical analysis

Statistical analysis was performed in R4.3.3 software, and MR Analysis was performed with the use of the “TwoSampleMR” package (Xu et al., 2019). During the MR analysis, we used Inverse Variance Weighted (IVW) as the main analysis method, and other supplementary analytical approaches included MR Egger, Weighted Median, Simple Mode, and Weighted Mode. Sensitivity analyses were conducted employing Cochran's *Q* statistic and MR-Egger regression to test for heterogeneity among SNP (*P* < 0.05 indicating heterogeneity), and MR-Egger regression to detect horizontal pleiotropy (*P* < 0.05 indicating pleiotropy). Leave-one-out sensitivity analysis was employed to assess whether any single SNP disproportionately influenced the causal relationship between gut microbiota and nonunion (Kou et al., 2020). In mediation analysis, a two-step MR approach was used to evaluate the mediating effects of immune cells between gut microbiota and nonunion (Bowden and Holmes, 2019; Evans and Davey Smith, 2015).

## Results

### Effect of GM on nonunion

**Figure 3:**
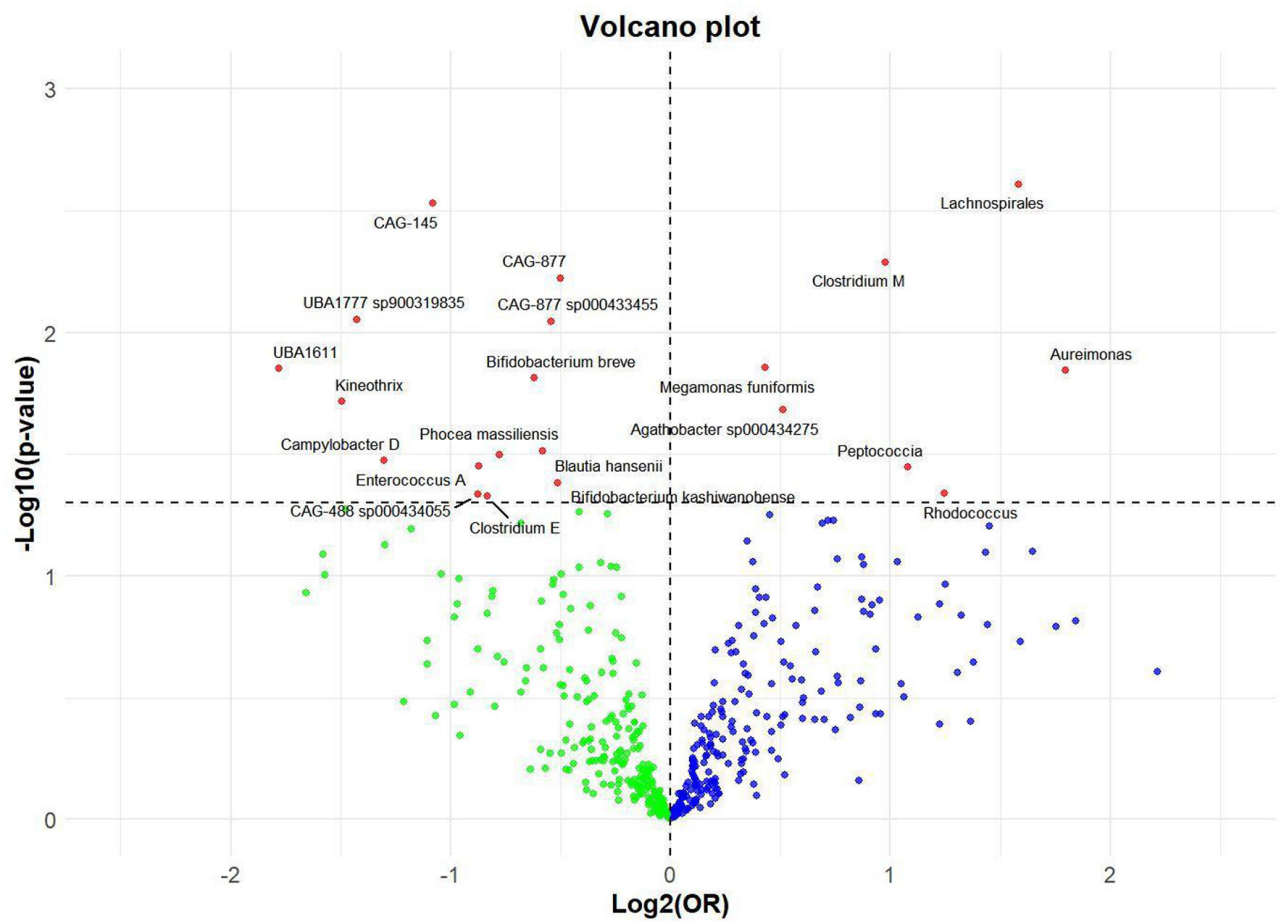
Volcano plot of correlations related to the influence of GM on nonunion. This plot includes both odds ratios (ORs) in log 2 scale and *P*-values in –log 10 estimated by the inverse variance weighted method for GWAS.

**Figure 4:**
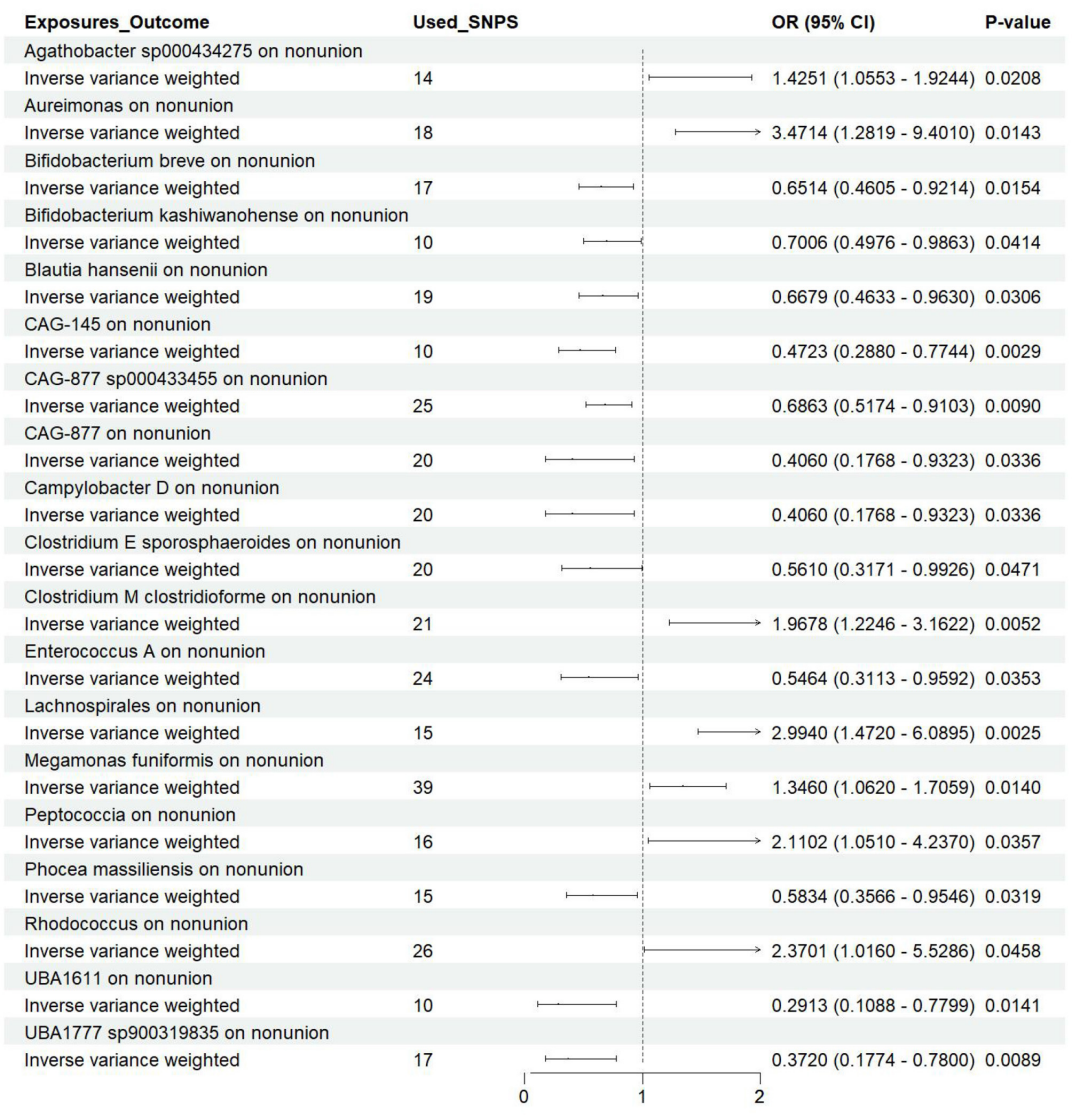
Forest plot of the causal effects of gut microbiota on the risk of nonunion derived from the IVW method. OR, odds ratio; CI, confidence interval.

**Figure 5:**
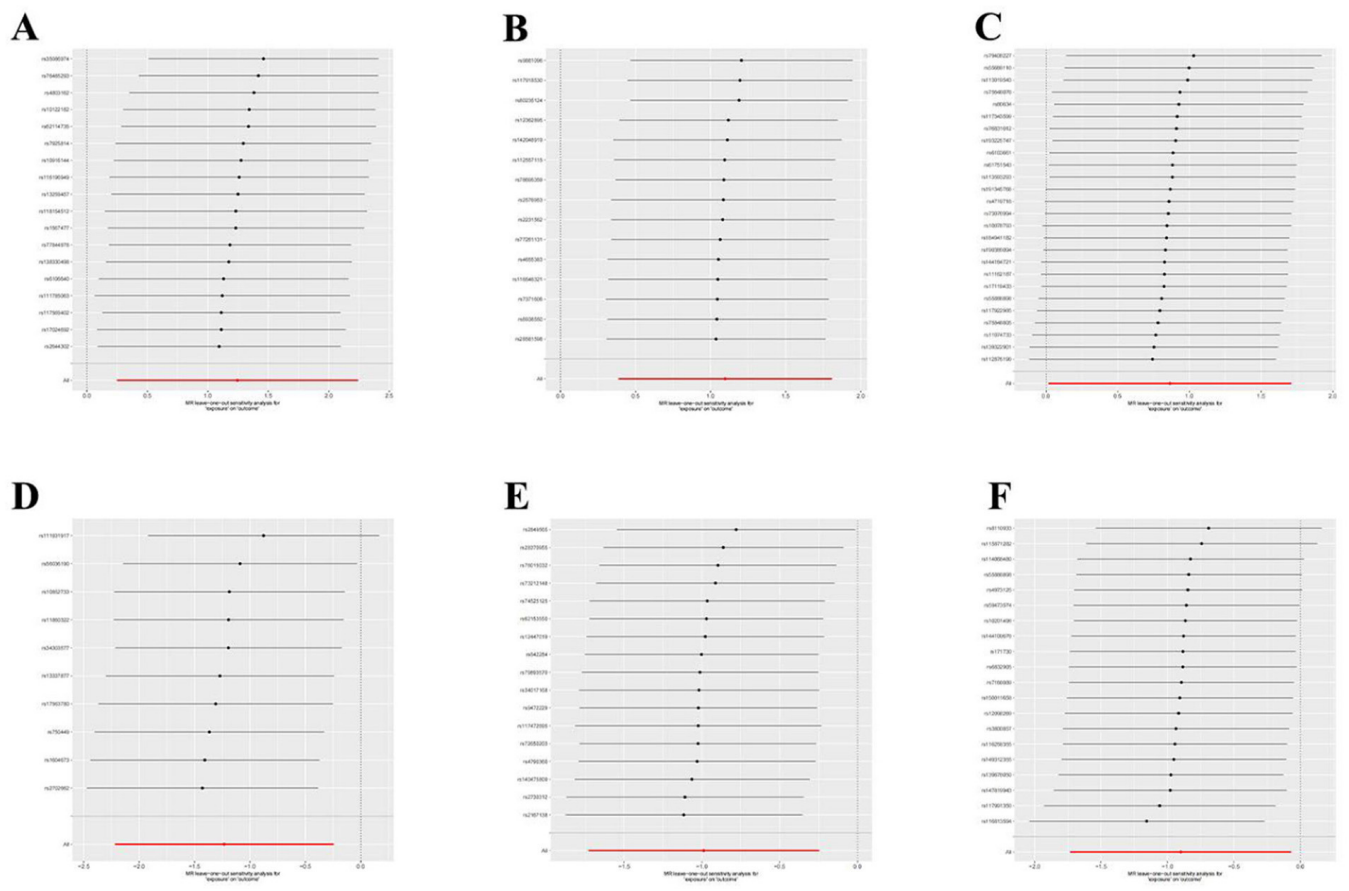
Leave-one-out plot to visualize the causal effect of each GM associated with nonunion when leaving one SNP out. **(A)** Aureimonas. **(B)** Lachnospirales. **(C)** Rhodococcus. **(D)** UBA1611. **(E)** UBA1777 sp900319835. **(F)** Campylobacter D.

Utilizing rigorous IV selection criteria, we identified 21 key gut microbiot that exhibit a significant causal relationship with nonunion. The causal effects of each gut microbiota on nonunion are detailed in Figures 3, 4 and Table 1. Of particular importance, among seven gut microbiota associated with an increased risk of nonunion, Aureimonas (OR = 1.968, 95% CI: 1.282–9.401, *P* = 0.014), Lachnospirales (OR = 2.994, 95% CI: 1.472–6.090, *P* = 0.002), and Rhodococcus (OR = 2.370, 95% CI: 1.016–5.529, *P* = 0.045) exhibited the most significant promoting effects. Conversely, among 12 gut microbiota associated with a decreased risk of nonunion, UBA1611 (OR = 0.291, 95% CI: 0.109–0.780, *P* = 0.014), UBA1777 sp900319835 (OR = 0.372, 95% CI: 0.177–0.780, *P* = 0.009), and Campylobacter D (OR = 0.406, 95% CI: 0.177–0.932, *P* = 0.034) demonstrated particularly significant preventive effects. The selected gut microbiota underwent Cochran's *Q*-test, which yielded *P*-values >0.05, suggesting no evidence of significant heterogeneity. Furthermore, the MR-Egger intercept test showed no statistical significance, indicating the absence of horizontal pleiotropy. Leave-one-out sensitivity analysis confirmed the robustness of the causal estimates, as removing specific SNPs did not alter the findings. Two gut microbiota species, CAG-488 sp000434055 (OR = 0.545, 95% CI: 0.300–0.990, *P* = 0.046) and Kineothrix (OR = 0.355, 95% CI: 0.149–0.845, *P* = 0.019), were excluded due to anomalies in pleiotropy or heterogeneity, despite their *P*-values being < 0.05. The remaining selected GMs showed *P*-values > 0.05 in Cochran *Q* tests, indicating no significant heterogeneity. The MR-Egger intercept test was not statistically significant, suggesting an absence of horizontal pleiotropy. Leave-one-out analysis confirmed the influence of each SNP on overall causal estimates, followed by a systematic reanalysis of the remaining SNPs (Figure 5, Supplementary Figure 1).

**Table 1:** The MR estimates of the causal relationships between 21 GM and the risk of nonunion and tests for heterogeneity and horizontal pleiotropy.

**Exposure**	**Methods**	**Number of SNPs**	***P*-value**	**OR**	**OR (95%CI)**		**Pleiotropy**	**Heterogeneity**	
					**Low**	**Upper**	***P*-value**	***Q*-value**	***P*-value**
Agathobacter sp000434275	IVW	14	0.021	1.425	1.055	1.924	0.290	7.419	0.829
Aureimonas	IVW	18	0.014	3.471	1.282	9.401	0.238	17.650	0.345
Bifidobacterium breve	IVW	17	0.015	0.651	0.460	0.921	0.582	15.260	0.433
Bifidobacterium kashiwanohense	IVW	10	0.041	0.701	0.498	0.986	0.396	8.048	0.429
Blautia hansenii	IVW	19	0.031	0.668	0.463	0.963	0.422	12.037	0.798
CAG-145	IVW	10	0.003	0.472	0.288	0.774	0.287	4.979	0.760
CAG-488 sp000434055	IVW	17	0.046	0.545	0.300	0.990	0.437	26.921	0.029
CAG-877 sp000433455	IVW	25	0.009	0.686	0.517	0.910	0.381	17.855	0.765
CAG-877	IVW	20	0.034	0.406	0.177	0.932	0.930	21.291	0.727
Campylobacter D	IVW	20	0.034	0.406	0.177	0.932	0.199	13.699	0.749
Clostridium E sporosphaeroides	IVW	20	0.047	0.561	0.317	0.993	0.806	5.344	0.998
Clostridium M clostridioforme	IVW	21	0.005	1.968	1.225	3.162	0.285	20.380	0.372
Enterococcus A	IVW	24	0.035	0.546	0.311	0.959	0.090	16.951	0.766
Kineothrix	IVW	17	0.019	0.355	0.149	0.845	0.027	10.083	0.815
Lachnospirales	IVW	15	0.002	2.994	1.472	6.090	0.744	5.223	0.970
Megamonas funiformis	IVW	39	0.014	1.346	1.062	1.706	0.381	37.603	0.441
Peptococcia	IVW	16	0.036	2.110	1.051	4.237	0.798	10.025	0.760
Phocea massiliensis	IVW	15	0.032	0.583	0.357	0.955	0.975	9.764	0.713
Rhodococcus	IVW	26	0.046	2.370	1.016	5.529	0.158	14.955	0.922
UBA1611	IVW	10	0.014	0.291	0.109	0.780	0.740	7.477	0.486
UBA1777 sp900319835	IVW	17	0.009	0.372	0.177	0.780	0.530	11.381	0.725

### Effect of immune cell traits on nonunion

**Figure 6:**
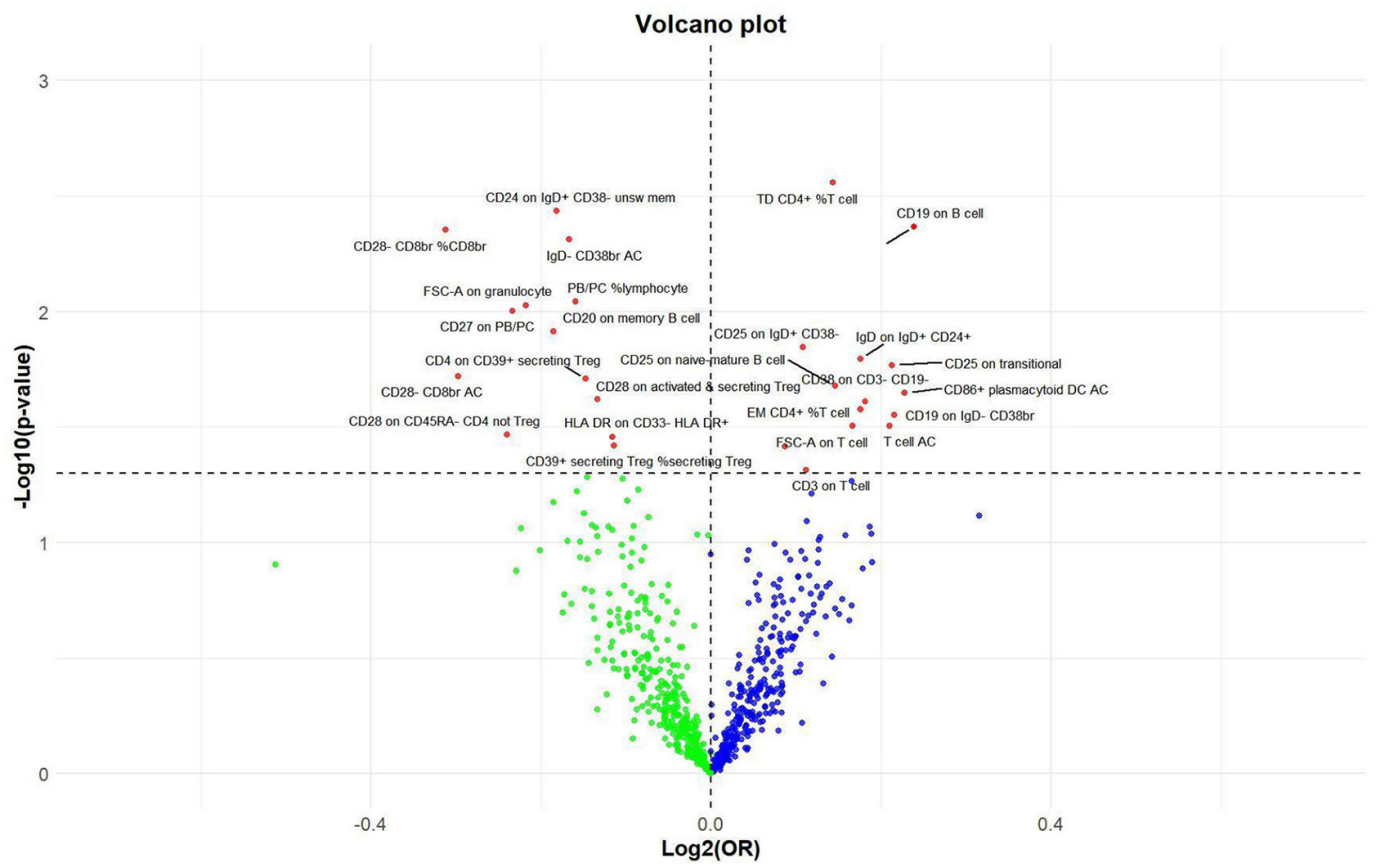
Volcano plot of correlations related to the influence of immune cell on nonunion. This plot includes both odds ratios (ORs) in log_2_ scale and *P*-values in –log_10_ estimated by the inverse variance weighted method for GWAS.

**Figure 7:**
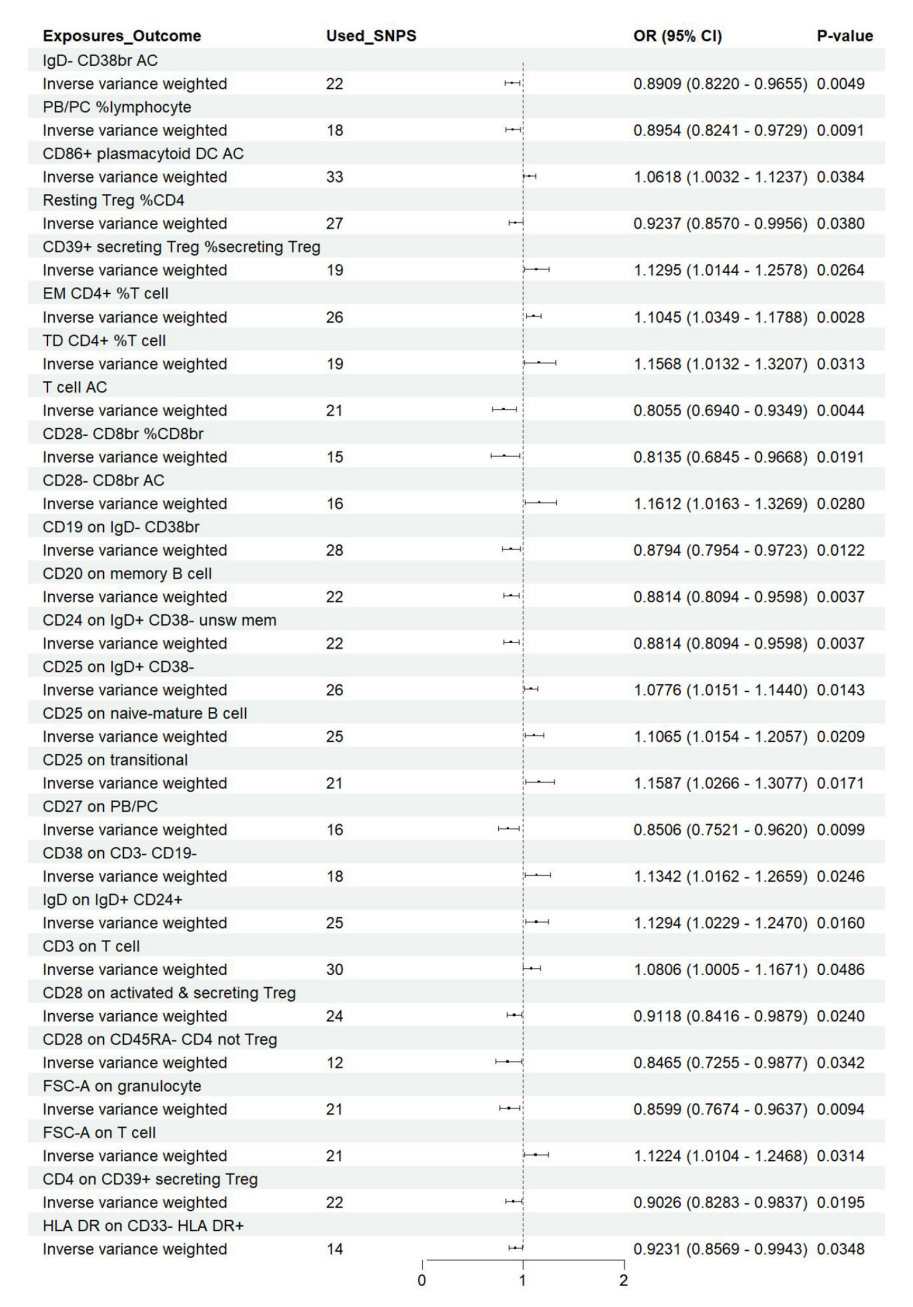
Forest plot of the causal effects of immune cell on the risk of nonunion derived from the IVW method. OR, odds ratio; CI, confidence interval.

**Figure 8:**
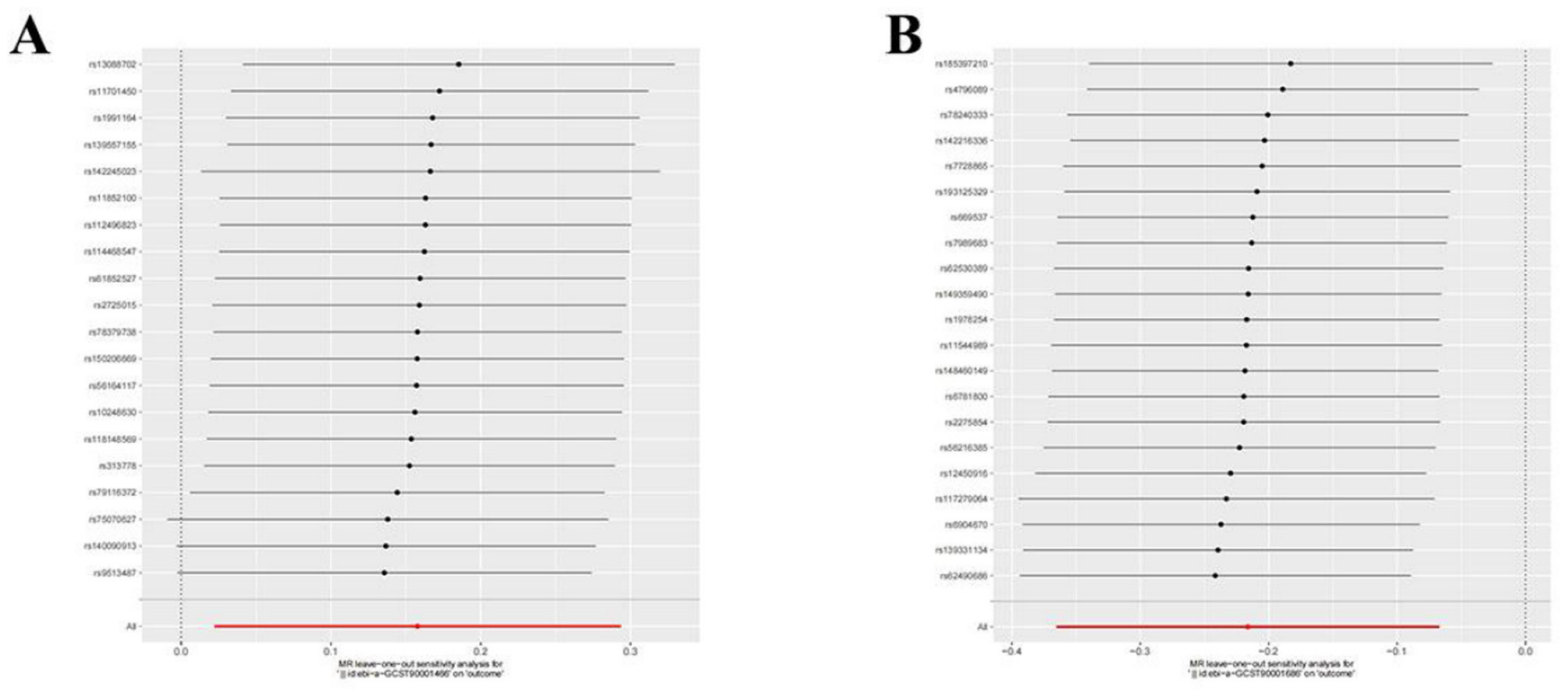
Leave-one-out plot to visualize the causal effect of each immune cell associated with nonunion when leaving one SNP out. **(A)** CD86+ plasmacytoid DC AC. **(B)** CD28- CD8br %CD8br.

Utilizing rigorous IV selection criteria, we identified 27 key immune cell traits that exhibit a significant causal relationship with nonunion. The causal effects of each immune cell traits on nonunion are detailed in Figures 6, 7 and Table 2. Twelve immune cell traits positively correlated with nonunion. Fourteen immune cell traits negatively correlated with nonunion. Notably, among the 14 immune cell types identified as risk factors for nonunion, the promoting effect of CD86+ plasmacytoid DC AC (OR = 1.171, 95% CI: 1.023–1.341, *P* = 0.022) was particularly pronounced. In contrast, within the 12 immune cell types associated with a reduced risk of nonunion, CD28– CD8br %CD8br (OR = 0.805, 95% CI: 0694–0.935, *P* = 0.004) exhibited the most significant preventive effect. CD19 on B cell (OR = 1.180, 95% CI: 1.053–1.323, *P* = 0.004), were excluded due to anomalies in pleiotropy, despite their *P*-values being < 0.05. The remaining selected GMs showed *P*-values > 0.05 in Cochran *Q* tests, indicating no significant heterogeneity. The MR-Egger intercept test was not statistically significant, suggesting an absence of horizontal pleiotropy. Leave-one-out analysis confirmed the influence of each SNP on overall causal estimates, followed by a systematic reanalysis of the remaining SNPs (Figure 8, Supplementary Figure 2).

**Table 2:** The MR estimates of the causal relationships between 27 immune cell traits and the risk of nonunion and tests for heterogeneity and horizontal pleiotropy.

**Exposure**	**Methods**	**Number of SNPs**	***P*-value**	**OR**	**OR (95%CI)**		**Pleiotropy**	**Heterogeneity**	
					**Low**	**Upper**	***P*** **-value**	***Q*** **-value**	***P*** **-value**
IgD– CD38br AC	IVW	22	0.005	0.891	0.822	0.966	0.590	19.849	0.467
PB/PC %lymphocyte	IVW	18	0.009	0.895	0.824	0.973	0.713	20.721	0.189
CD86+ plasmacytoid DC AC	IVW	20	0.022	1.171	1.023	1.341	0.582	11.815	0.857
Resting Treg %CD4	IVW	33	0.038	1.062	1.003	1.124	0.472	24.715	0.817
CD39+ secreting Treg %secreting Treg	IVW	27	0.038	0.924	0.857	0.996	0.411	14.069	0.961
EM CD4+ %T cell	IVW	19	0.026	1.130	1.014	1.258	0.105	7.345	0.834
TD CD4+ %T cell	IVW	26	0.003	1.104	1.035	1.179	0.629	24.308	0.444
T cell AC	IVW	19	0.031	1.157	1.013	1.321	0.197	14.556	0.627
CD28– CD8br %CD8br	IVW	21	0.004	0.805	0.694	0.935	0.129	11.428	0.909
CD28– CD8br AC	IVW	15	0.019	0.814	0.685	0.967	0.600	9.456	0.738
CD19 on IgD– CD38br	IVW	16	0.028	1.161	1.016	1.327	0.574	15.716	0.331
CD20 on memory B cell	IVW	28	0.012	0.879	0.795	0.972	0.858	27.118	0.403
CD24 on IgD+ CD38– unsw mem	IVW	22	0.004	0.881	0.809	0.960	0.148	14.496	0.804
CD25 on IgD+ CD38–	IVW	26	0.014	1.078	1.015	1.144	0.178	18.822	0.761
CD25 on naive-mature B cell	IVW	25	0.021	1.106	1.015	1.206	0.247	15.317	0.883
CD25 on transitional	IVW	21	0.017	1.159	1.027	1.308	0.454	14.312	0.765
CD27 on PB/PC	IVW	16	0.010	0.851	0.752	0.962	0.190	12.318	0.581
CD38 on CD3– CD19–	IVW	18	0.025	1.134	1.016	1.266	0.335	14.335	0.574
IgD on IgD+ CD24+	IVW	25	0.016	1.129	1.023	1.247	0.452	15.320	0.883
CD3 on T cell	IVW	30	0.049	1.081	1.000	1.167	0.692	19.436	0.884
CD28 on activated & secreting Treg	IVW	12	0.034	0.846	0.725	0.988	0.212	10.870	0.368
CD28 on CD45RA– CD4 not Treg	IVW	12	0.034	0.846	0.725	0.988	0.212	11.000	0.306
FSC-A on granulocyte	IVW	21	0.009	0.860	0.767	0.964	0.748	18.678	0.478
FSC-A on T cell	IVW	21	0.031	1.122	1.010	1.247	0.561	25.961	0.131
CD19 on B cell	IVW	24	0.0043	1.1804	1.0534	1.3227	0.0300	17.5256	0.7337
CD4 on CD39+ secreting Treg	IVW	22	0.020	0.903	0.828	0.984	0.544	10.392	0.960

### Effect of GM on immune cell traits

**Figure 9:**
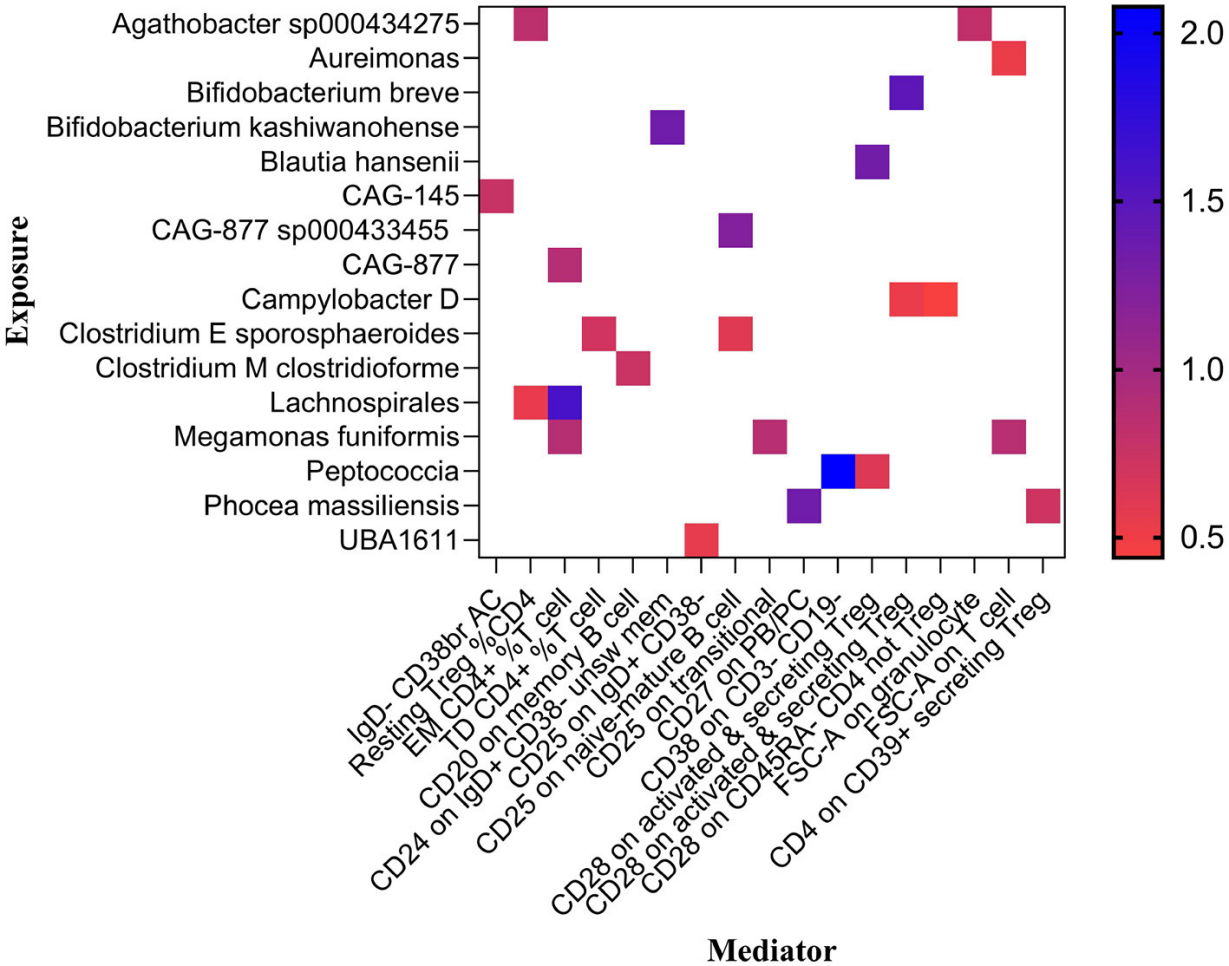
Mendelian randomization analysis between GM and Mediator.

Previously, we identified 19 genera and 26 immune cell traits on nonuion. Then, we investigated the causal role of 19 genera on 26 immune cell traits. The MR analysis revealed that 24 different GM and immune cell combinations that causally impact nonunion (Figure 9 and Table 3). At the same time, No heterogeneity and horizontal pleiotropy were observed, and a particular SNP did not drive causal estimates (Table 3).

**Table 3:** Mendelian randomization analysis between Microbiota and Mediator.

**Exposure**	**Outcome**	**Number of SNPs**	***P*-value**	**OR**	**OR (95%CI)**		**Pleiotropy**	**Heterogeneity**	
					**Low**	**Upper**	***P*** **-value**	***Q*** **-value**	***P*** **-value**
Lachnospirales	Resting Treg %CD4	14	0.0031	0.5488	0.3688	0.8166	0.6705	12.3942	0.4146
CAG-145	IgD- CD38br AC	10	0.0230	0.7579	0.5967	0.9626	0.5007	6.6585	0.5739
Agathobacter sp000434275	Resting Treg %CD4	14	0.0326	0.8321	0.7030	0.9849	0.9769	13.5440	0.3308
CAG-877	EM CD4+ %T cell	28	0.0496	0.8799	0.7744	0.9998	0.6310	23.8439	0.5849
Lachnospirales	EM CD4+ %T cell	14	0.0207	1.6016	1.0747	2.3869	0.1048	7.3449	0.8340
Megamonas funiformis	EM CD4+ %T cell	38	0.0276	0.8710	0.7702	0.9849	0.5860	33.5136	0.5874
Clostridium E sporosphaeroides	TD CD4+ %T cell	20	0.0497	0.7002	0.4906	0.9995	0.1551	11.2985	0.8813
Clostridium M clostridioforme	CD20 on memory B cell	23	0.0268	0.7449	0.5740	0.9667	0.1646	16.7288	0.7274
Bifidobacterium kashiwanohense	CD24 on IgD+ CD38- unsw mem	12	0.0348	1.3463	1.0214	1.7745	0.8526	7.3919	0.6880
UBA1611	CD25 on IgD+ CD38-	12	0.0413	0.5550	0.3152	0.9771	0.5421	1.3963	0.9992
CAG-877 sp000433455	CD25 on naive-mature B cell	24	0.0221	1.2166	1.0286	1.4391	0.7925	24.9509	0.2994
Clostridium E sporosphaeroides	CD25 on naive-mature B cell	20	0.0076	0.5990	0.4112	0.8726	0.8113	20.5416	0.3032
Megamonas funiformis	CD25 on transitional	37	0.0499	0.8674	0.7524	1.0000	0.3949	42.5127	0.1790
Phocea massiliensis	CD27 on PB/PC	15	0.0306	1.3412	1.0279	1.7501	0.7565	13.0729	0.4422
Peptococcia	CD38 on CD3- CD19-	15	0.0216	2.0781	1.1132	3.8792	0.0541	11.8285	0.5418
Blautia hansenii	IgD on IgD+ CD24+	19	0.0193	1.3266	1.0469	1.6810	0.9591	11.7152	0.8171
Peptococcia	IgD on IgD+ CD24+	16	0.0267	0.6230	0.4100	0.9468	0.3582	6.5008	0.9522
Bifidobacterium breve	CD28 on activated & secreting Treg	16	0.0042	1.4641	1.1275	1.9012	0.1078	11.1424	0.6748
Campylobacter D	CD28 on activated & secreting Treg	19	0.0222	0.5203	0.2973	0.9108	0.3049	18.7688	0.3420
Campylobacter D	CD28 on CD45RA- CD4 not Treg	19	0.0033	0.4405	0.2551	0.7609	6.4666	0.9895	0.9007
Agathobacter sp000434275	FSC-A on granulocyte	14	0.0383	0.8109	0.6651	0.9888	0.4434	9.6321	0.6482
Aureimonas	FSC-A on T cell	17	0.0452	0.5321	0.2870	0.9866	0.3158	13.7818	0.5421
Megamonas funiformis	FSC-A on T cell	38	0.0293	0.8657	0.7604	0.9856	0.2921	27.3471	0.8496
Phocea massiliensis	CD4 on CD39+ secreting Treg	15	0.0347	0.7231	0.5352	0.9770	0.8023	10.3002	0.6692

### A reverse MR analysis

We identified causal relationships between 21 gut microbiota with significant characteristics and nonunion. Subsequently, we performed a reverse Mendelian Randomization analysis, which revealed no reverse causal effects of nonunion on the 21 gut microbiota. No heterogeneity and horizontal pleiotropy were discovered (Table 4).

**Table 4:** A reverse MR analysis showed no causal role of nonunion on 21 GM.

**Outcome**	**Methods**	**Number of SNPs**	***P*-value**	**OR**	**OR (95% CI)**		**Pleiotropy**	**Heterogeneity**	
					**Low**	**Upper**	***P*** **-value**	***Q*** **-value**	***P*** **-value**
Agathobacter sp000434275	482.0000	0.4863	0.9973	0.9897	1.0049	0.8505	449.6752	0.8361	IVW
Aureimonas	482.0000	0.4091	1.0009	0.9988	1.0029	0.7247	516.3562	0.1217	IVW
Bifidobacterium breve	482.0000	0.3453	1.0023	0.9975	1.0072	0.4703	522.3973	0.0883	IVW
Bifidobacterium kashiwanohense	482.0000	0.5355	1.0021	0.9955	1.0087	0.8843	523.2214	0.084	IVW
Blautia hansenii	482.0000	0.1840	0.9969	0.9924	1.0015	0.7918	580.1170	0.001	IVW
CAG-145	482.0000	0.2625	1.0027	0.9980	1.0074	0.9989	533.8749	0.045	IVW
CAG-488 sp000434055	482.0000	0.9343	1.0002	0.9959	1.0045	0.0727	456.7359	0.771	IVW
CAG-877 sp000433455	482.0000	0.1779	1.0036	0.9984	1.0088	0.2528	499.7008	0.258	IVW
CAG-877	482.0000	0.2443	1.0034	0.9977	1.0092	0.2360	497.9795	0.276	IVW
Campylobacter D	482.0000	0.2561	0.9988	0.9967	1.0009	0.8669	539.1566	0.034	IVW
Clostridium E sporosphaeroides	482.0000	0.7316	1.0005	0.9976	1.0034	0.7143	520.1054	0.100	IVW
Clostridium M clostridioforme	482.0000	0.2969	0.9980	0.9943	1.0017	0.2802	517.5266	0.115	IVW
Enterococcus A	482.0000	0.2728	0.9987	0.9964	1.0010	0.5299	515.7503	0.126	IVW
Kineothrix	482.0000	0.0965	0.9980	0.9956	1.0004	0.8827	512.2802	0.149	IVW
Lachnospirales	482.0000	0.0741	0.9972	0.9941	1.0003	0.8291	534.5492	0.043	IVW
Megamonas funiformis	482.0000	0.5221	1.0016	0.9968	1.0063	0.0075	469.1238	0.630	IVW
Peptococcia	482.0000	0.8716	1.0002	0.9979	1.0025	0.1239	462.3589	0.711	IVW
Phocea massiliensis	482.0000	0.7701	0.9994	0.9956	1.0033	0.7430	498.8575	0.267	IVW
Rhodococcus	482.0000	0.1061	1.0013	0.9997	1.0030	0.2335	490.7729	0.357	IVW
UBA1611	482.0000	0.7395	0.9996	0.9973	1.0019	0.4782	445.7281	0.867	IVW
UBA1777 sp900319835	482.0000	0.1702	1.0019	0.9992	1.0045	0.3183	484.8903	0.429	IVW

### Mediation effect of GM on nonunion

**Figure 10:**
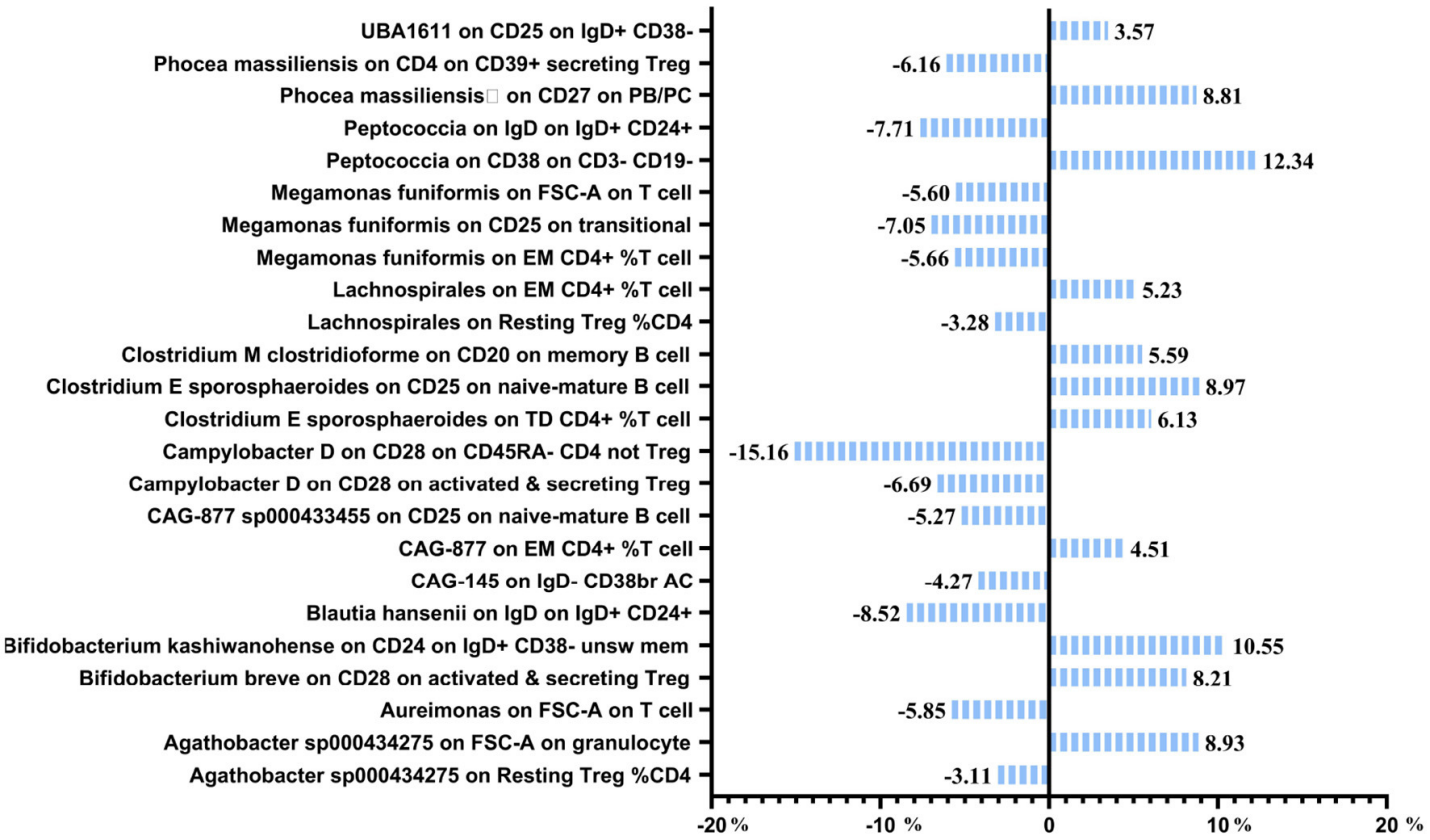
Mediation analysis of immune cell trait between GM and nonunion.

The previous Mendelian Randomization (MR) analysis between gut microbiota (GM) and nonunion revealed that certain bacterial families exhibited significant associations with the risk of nonunion. Specifically, Aureimonas, Lachnospirales, and Rhodococcus were identified as significantly promoting the occurrence of nonunion. In contrast, UBA1611, UBA1777 sp900319835, and Campylobacter D were significantly associated with reduced risk, suggesting a potential protective role. To better understand these relationships, we conducted a mediation analysis to investigate the involvement of immune cell characteristics in mediating the effects of GM on nonunion. The mediation MR analysis demonstrated that the family Aureimonas exerted its effects on nonunion through FSC-A on T cells, with a mediation proportion of 5.852%. Notably, FSC-A on T cells was associated with an increased risk of nonunion, indicating a detrimental role in bone healing. The family Lachnospirales mediated its influence on nonunion through Resting Treg %CD4 and EM CD4+ %T cells, with mediation proportions of 3.279% and 5.232%, respectively. Both Resting Treg %CD4 and EM CD4+ %T cells were positively correlated with an increased risk of nonunion, suggesting that these immune cell subsets might contribute to delayed or impaired bone healing. Interestingly, no significant mediation effects were observed between immune cells and the families Rhodococcus or UBA1777 sp900319835 in relation to nonunion, suggesting that their influence may be independent of immune modulation. The family UBA1611 mediated its effects on nonunion through CD25 on IgD+ CD38–, with a mediation proportion of 3.569%. CD25 on IgD+ CD38– was also linked to an increased risk of nonunion, further supporting its potential role in hindering bone regeneration. The family Campylobacter D mediated its protective effects on nonunion through CD28 on CD45RA- CD4 not Treg and CD28 on activated & secreting Treg, with mediation proportions of 15.156% and 6.688%, respectively. Both CD28 on CD45RA– CD4 not Treg and CD28 on activated & secreting Treg were associated with a reduced risk of nonunion, indicating that these immune markers might facilitate effective immune regulation and bone healing. The Bifidobacterium breve and Bifidobacterium kashiwanohense exhibited mediation effects via CD28 on activated & secreting Treg (8.207%) and CD24 on IgD+ CD38– unsw mem (10.551%). Moreover, additional mediation analyses of other microbiota-immune cell interactions highlighted further modulation of inflammatory pathways. Key immune cell populations, including regulatory T cells and memory B cells, emerged as critical mediators in these microbiota-immune interactions, ultimately affecting bone healing outcomes (Figure 10 and Table 5). These findings provide insights into the complex interplay between gut microbiota, immune cells, and bone healing. Understanding these interactions could pave the way for targeted microbiome-based therapies aimed at promoting bone regeneration and preventing nonunion.

**Table 5:** Mediation effect of GM on nonunion via immune cell.

**Exposure**	**Outcome**	**Beta (B) beta_all**	**beta1**	**beta2**	**beta12**	**Beta (A) beta_dir**	**Mediated Proportion (%)**
Lachnospirales	Resting Treg %CD4	1.097	−0.600	0.060	−0.036	1.133	−3.279%
CAG-145	IgD- CD38br AC	−0.750	−0.277	−0.116	0.032	−0.782	−4.271%
Agathobacter sp000434275	Resting Treg %CD4	0.354	−0.184	0.060	−0.011	0.365	−3.109%
CAG-877	EM CD4+ %T cell	−0.346	−0.128	0.122	−0.016	−0.330	4.508%
Lachnospirales	EM CD4+ %T cell	1.097	0.471	0.122	0.057	1.039	5.232%
Megamonas funiformis	EM CD4+ %T cell	0.297	−0.138	0.122	−0.017	0.314	−5.664%
Clostridium E sporosphaeroides	TD CD4+ %T cell	−0.578	−0.356	0.099	−0.035	−0.543	6.126%
Clostridium M clostridioforme	CD20 on memory B cell	0.677	−0.295	−0.128	0.038	0.639	5.591%
Bifidobacterium kashiwanohense	CD24 on IgD+ CD38– unsw mem	−0.356	0.297	−0.126	−0.038	−0.318	10.551%
UBA1611	CD25 on IgD+ CD38–	−1.233	−0.589	0.075	−0.044	−1.189	3.569%
CAG-877 sp000433455	CD25 on naive-mature B cell	−0.376	0.196	0.101	0.020	−0.396	−5.272%
Clostridium E sporosphaeroides	CD25 on naive-mature B cell	−0.578	−0.513	0.101	−0.052	−0.526	8.974%
Megamonas funiformis	CD25 on transitional	0.297	−0.142	0.147	−0.021	0.318	−7.051%
Phocea massiliensis	CD27 on PB/PC	−0.539	0.294	−0.162	−0.048	−0.491	8.815%
Peptococcia	CD38 on CD3– CD19–	0.747	0.731	0.126	0.092	0.655	12.336%
Blautia hansenii	IgD on IgD+ CD24+	−0.404	0.283	0.122	0.034	−0.438	−8.525%
Peptococcia	IgD on IgD+ CD24+	0.747	−0.473	0.122	−0.058	0.804	−7.712%
Bifidobacterium breve	CD28 on activated & secreting Treg	−0.429	0.381	−0.092	−0.035	−0.393	8.207%
Campylobacter D	CD28 on activated & secreting Treg	−0.901	−0.653	−0.092	0.060	−0.962	−6.688%
Campylobacter D	CD28 on CD45RA– CD4 not Treg	−0.901	−0.820	−0.167	0.137	−1.038	−15.156%
Agathobacter sp000434275	FSC-A on granulocyte	0.354	−0.210	−0.151	0.032	0.323	8.926%
Aureimonas	FSC-A on T cell	1.245	−0.631	0.115	−0.073	1.317	−5.852%
Megamonas funiformis	FSC-A on T cell	0.297	−0.144	0.115	−0.017	0.314	−5.603%
Phocea massiliensis	CD4 on CD39+ secreting Treg	−0.539	−0.324	−0.102	0.033	−0.572	−6.162%

Beta B (total effect): The causal role of GM on nonunion. beta1: The causal role of GM on immune cell traits. beta2: the causal role of immune cell traits on nonunion. Beta A (Direct effect) = β(beta1) ^*^ β(beta2). Indirect effect = Beta B-Beta A. The mediated proportion = Beta A/Beta B.

## Discussion

The intricate relationship between gut microbiota and immune system-mediated diseases has garnered significant attention in recent years. This complex interplay significantly influences health and disease, where disruptions in this balance can lead to immune dysregulation (Zheng et al., 2020; Xu et al., 2019). Previous observational studies have extensively explored the relationships between phenotypes and diseases. However, these studies often encounter confounding factors related to instrumentation, equipment, procedures, and sampling, limiting their utility for establishing causal relationships (Kou et al., 2020). Consequently, although previous observational studies have suggested an association between gut microbiota and bone health, potential confounders and reverse causality have precluded definitive causal inferences (Lu et al., 2021). To our knowledge, this is the first study to establish causal relationships between gut microbiota, immune cells, and nonunion using mediated Mendelian randomization analysis. Our study employed Mendelian randomization to thoroughly investigate the associations between GM and nonunion, offering compelling insights into these complex interactions. We identified a positive correlation between Agathobacter sp000434275, Aureimonas, Clostridium M, Lachnospirales, Megamonas funiformis, Peptococcia, and Rhodococcus and nonunion. This suggests that an increased abundance of these taxa may be linked to a higher risk of nonunion. Conversely, Bifidobacterium breve, Bifidobacterium kashiwanohense, Blautia hansenii, CAG-145, CAG-877 sp000433455, CAG-877, Campylobacter D, Clostridium E, Enterococcus A, Phocea massiliensis, UBA1611, and UBA1777 sp900319835 are negatively correlated with nonunion. This indicates that these taxa may exert protective effects against the condition. Our findings highlight specific gut microbiota changes that could influence the risk of nonunion. This nuanced understanding underscores that the human body is an interconnected system with distinct parts. Our findings indicate that gut microbiota, including Aureimonas and Lachnospirales, may increase the risk of nonunion, whereas Campylobacter D and Bifidobacterium appear to have a protective effect. However, the specific pathways and mechanisms through which these gut microbiota impact nonunion remain unclear.

Gut microbiota are believed to impact bone metabolism through three primary mechanisms: regulating nutrient absorption, modulating the host immune system, and translocating microbial products across the gut epithelial barrier (Seely et al., 2021). Probiotics can enhance mineral absorption and bone health in the gut, either directly or by regulating bile acid metabolism and vitamin production (Rodríguez et al., 2013). Additionally, microbial products, termed microbe-associated molecular patterns (MAMPs), can cross the gut epithelial barrier and enter the systemic circulation. More importantly, gut bacteria can influence bone health by modulating the immune system. Activation of immune cells in the gut can release cytokines into the systemic circulation or migrate to bones, directly affecting the activity of osteoblasts and osteoclasts. Additionally, gut microbiota can stimulate systemic inflammatory processes, activating innate immune receptors on osteoclasts and osteoblasts, thus influencing bone remodeling (Hernandez, 2017).

Immune cells play a crucial role in fracture healing, with the final outcome highly dependent on the initial inflammatory phase. Furthermore, immune cells and bone marrow-derived mesenchymal stem cells (bm-MSCs) engage in critical intercellular communication or crosstalk to modulate bone healing. Inflammation marks the early response to fractures, similar to other tissue injuries (Maruyama et al., 2020). Successful healing depends on the initiation of a robust inflammatory response involving various cell types (Bahney et al., 2019; Claes et al., 2012). T cells are crucial during the cartilage and osseous callus stages of healing. Studies indicate that T cells are selectively recruited to fracture sites during the early repair phase and remain at the injury site until the later stages of healing (McHugh, 2023; Könnecke et al., 2014). Consequently, their numbers increase disproportionately at fracture sites compared to non-fracture areas. The beneficial role of T cells is underscored by the higher incidence of nonunion in patients treated with immunosuppressants and in those with AIDS compared to healthy individuals (Aurora and Silva, 2023; Bissinger et al., 2016). However, because B cells regulate T cells through antigen presentation and the CD40/CD40L system, T cells may only promote fracture healing in the presence of B cells (Askalonov et al., 1987; Dar et al., 2023). Animal studies have confirmed that a reduction in B cell numbers stimulates fracture healing. Subsequently, as osteoblast numbers increase during the callus reconstruction phase, B cell numbers gradually return, limiting additional bone regeneration (Zhang et al., 2021). Our findings indicate that various T cell types, including CD28- CD8br %CD8br, CD28-CD8br AC, CD28 on activated & secreting Treg, and CD4 on CD39+ secreting Treg, confer protective effects against nonunion. Conversely, T cell types such as TD CD4+ %T cell, FSC-A on T cell, and EM CD4+ %T cell are associated with higher risks of nonunion. Meanwhile, B cell types such as CD20 on memory B cell, CD24 on IgD+ CD38- unsw mem, and PB/PC %lymphocyte exhibit protective effects against nonunion. In contrast, B cell types such as CD25 on naive-mature B cell, IgD on IgD+ CD24+, CD38 on CD3-CD19-, CD25 on transitional, and CD19 on IgD- CD38br are associated with higher risks of nonunion.

Immune system dysregulation is often associated with alterations in GM, leading to diseases such as osteoporosis and rheumatoid arthritis. Understanding the dynamic interactions between GM, immune cell traits, and bone metabolism could facilitate the development of targeted interventions to promote bone health (Minetti et al., 2022; Contaldo et al., 2021; Inchingolo et al., 2021). Our mediation MR analysis revealed that various types of gut microbiota can influence nonunion through different immune cell traits. Specifically, Bifidobacterium influences nonunion through CD28 on activated & secreting Treg and CD24 on IgD+ CD38- unsw mem, achieving a combined mediation efficacy of 18.7%. Megamonas funiformis affects nonunion through CD25 on transitional, achieving a combined mediation efficacy of 18.3%. Clostridium E sporosphaeroides influences nonunion through TD CD4+ %T cell and CD25 on naive-mature B cell, achieving a combined mediation efficacy of 15.1%. Notably, the regulation of nonunion by Agathobacter sp000434275, CAG-877, Lachnospirales, Peptococcia, and Phocea massiliensis is bidirectionally mediated by immune cells. This suggests a consistent relationship between specific gut bacteria and immune cell dynamics in patients with nonunion. This emphasizes the significant impact of specific gut microbiota on systemic immune cell behavior and highlights the potential of these quantifiable relationships as targets for early screening, disease assessment, and therapeutic interventions.

Currently, most studies investigating the relationship between GM and nonunion are observational, leaving the genetic causal relationship between them unclear. To our knowledge, this study is the first to use SNPs as instrumental variables to exclude potential confounders and effectively establish the causal relationship between GM and nonunion, along with the mediating role of immune cells, using Mendelian Randomization (MR) analysis. A notable feature of our research is the detailed exploration of specific gut microbiota genera and their associations with nonunion. These findings offer intriguing insights into potential biological interactions. Additionally, our data comes from newly identified data on 473 gut microbiota and nonunion GWAS data. Moreover, our methodology integrates multiple rigorous analytical models, enhancing the robustness of our conclusions. Additionally, given the autoimmune nature of nonunion, investigating specific microbial determinants can uncover key pathways and intervention points, paving the way for innovative therapies and personalized strategies for treating nonunion.

This study uniquely utilizes mediation Mendelian randomization analysis to elucidate the causal relationships between gut microbiota, immune cells, and nonunion. While specific gut microbiota associated with an increased or decreased risk of nonunion were identified, gaining insight into the pathways and key risk genes involved is crucial for formulating future strategies aimed at modifying nonunion prevention and treatment through targeted gut microbiota interventions. Identifying the critical factors in this process is a major focus of our team's future research initiatives. Our research aims to further explore individual-level heterogeneity and apply nonlinear analyses to unravel the complex relationships between the microbiome and immunity. Concentrating on the gut microbiota-immune cell-nonunion axis, we seek to develop a pathway-based polygenic regression approach, integrating GWAS summary statistics and scRNA-seq data to pinpoint trait-related individual cells and assess their causal relationship with nonunion. Future plans involve the integration of scRNA-seq data from nonunion and immune cells, utilizing the newly developed scPagwas method to identify immune cell subpopulations, specific pathways, and key genes linked to nonunion traits (Ma et al., 2022, 2023). By employing microbial co-occurrence networks in combination with multi-omics techniques, we aim to explore microbial abundance patterns and their interactions with immune markers. Moreover, dynamic time-series analysis will be employed to examine temporal associations between key microbes and immune functions, ultimately providing further insights into critical targets within the gut microbiota-immune cell-nonunion axis and establishing a foundation for clinical applications.

However, our study has several limitations. Firstly, although the use of SNPs as instrumental variables eliminated confounders and enhanced the accuracy and reliability of our conclusions, important covariates such as environment, lifestyle, surgical methods, and medication use could not be adjusted. Secondly, MR analysis typically reveals lifelong exposure scenarios, necessitating further investigation through randomized controlled trials. Given that most publicly available studies on the gut microbiome and nonunion are cross-sectional, longitudinal studies would offer more robust validation of causal relationships. Future research should prioritize longitudinal designs to enhance the reliability of findings and facilitate their translation into clinical practice. Furthermore, publicly available genetic data related to the gut microbiome and nonunion predominantly originate from European populations, which may introduce bias into the current study. To ensure the generalizability of the findings, future research should aim to incorporate data from diverse populations, including those of Asian, African, and other ancestries. Finally, the lack of individual-level data limited our exploration of more complex relationships, potentially overlooking nonlinear associations between gut microbiota, immune cells, and nonunion.

## Conclusion

Our mediation Mendelian Randomization (MR) analysis has revealed key causal relationships and associated risks among gut microbiota, immune cells, and nonunion. By identifying specific microbial taxa and immune cell characteristics that influence nonunion. Considering the adaptability of gut microbiota, dietary interventions emerge as a promising therapeutic strategy. Investigating the potential of cultivating protective microbial taxa through specific diets could revolutionize the management of nonunion. Fundamentally, the integration of basic science, clinical insights, and advanced analytical tools enhances our understanding of the complex interactions among gut microbiota, nonunion, and the immune system. Exploring specific microbial determinants may reveal key pathways and intervention points for nonunion, paving the way for innovative therapies and personalized strategies, and offering substantial hope for transforming the treatment landscape.

## Data Availability

The original contributions presented in the study are included in the article/Supplementary material, further inquiries can be directed to the corresponding author.

## Supplementary material

Supplementary Figure 1 Leave-one-out plot to visualize the causal effect of each GM associated with nonunion when leaving one SNP out. **(A)** Agathobacter sp000434275. **(B)** Aureimonas. **(C)** *Bifidobacterium breve*. **(D)** *Bifidobacterium kashiwanohense*. **(E)** *Blautia hansenii*. **(F)** CAG-145. **(G)** CAG-488 sp000434055. **(H)** CAG-877 sp000433455. **(I)** CAG-877. **(J)** Campylobacter D. **(K)**. Clostridium E. **(L)** Clostridium. **(M)** Enterococcus A. **(N)** Kineothrix. **(O)** Lachnospirales. **(P)** Megamonas funiformis. **(Q)** Peptococcia. **(R)** *Phocea massiliensis*. **(S)** Phocea massiliensis. **(T)** *Phocea massiliensis*. **(U)** Rhodococcus. **(V)** UBA1777 sp900319835.

Supplementary Figure 2 Leave-one-out plot to visualize the causal effect of each immune cell associated with nonunion when leaving one SNP out. **(A)** IgD– CD38br AC. **(B)** PB/PC %lymphocyte. **(C)** CD86+ plasmacytoid DC AC. **(D)** Resting Treg %CD4. **(E)** CD39+ secreting Treg %secreting Treg. **(F)** EM CD4+ %T cell. **(G)** TD CD4+ %T cell. **(H)** T cell AC. **(I)** CD28– CD8br %CD8br. **(J)** CD28– CD8br AC. **(K)** CD19 on IgD– CD38br. **(L)** CD20 on memory B cell. **(M)** CD24 on IgD+ CD38– unsw mem. **(N)** CD25 on IgD+ CD38–. **(O)** CD25 on naive-mature B cell. **(P)** CD25 on transitional. **(Q)** CD27 on PB/PC. **(R)** CD38 on CD3– CD19–. **(S)** IgD on IgD+ CD24+. **(T)** CD3 on T cell. **(U)** CD28 on activated & secreting Treg. **(V)** CD28 on CD45RA– CD4 not Treg. **(W)** FSC-A on granulocyte. **(X)** FSC-A on T cell. **(Y)** CD4 on CD39+ secreting Treg. **(Z)** HLA DR on CD33– HLA DR+.
