# Supplementary figures and images for: Exploring the causal relationship of gut microbiota in nonunion: a Mendelian randomization analysis mediated by immune cell
Source: Front Microbiol. 2024 Dec 16;15:1447877. doi: 10.3389/fmicb.2024.1447877 (PMC11683590; doi:10.3389/fmicb.2024.1447877)

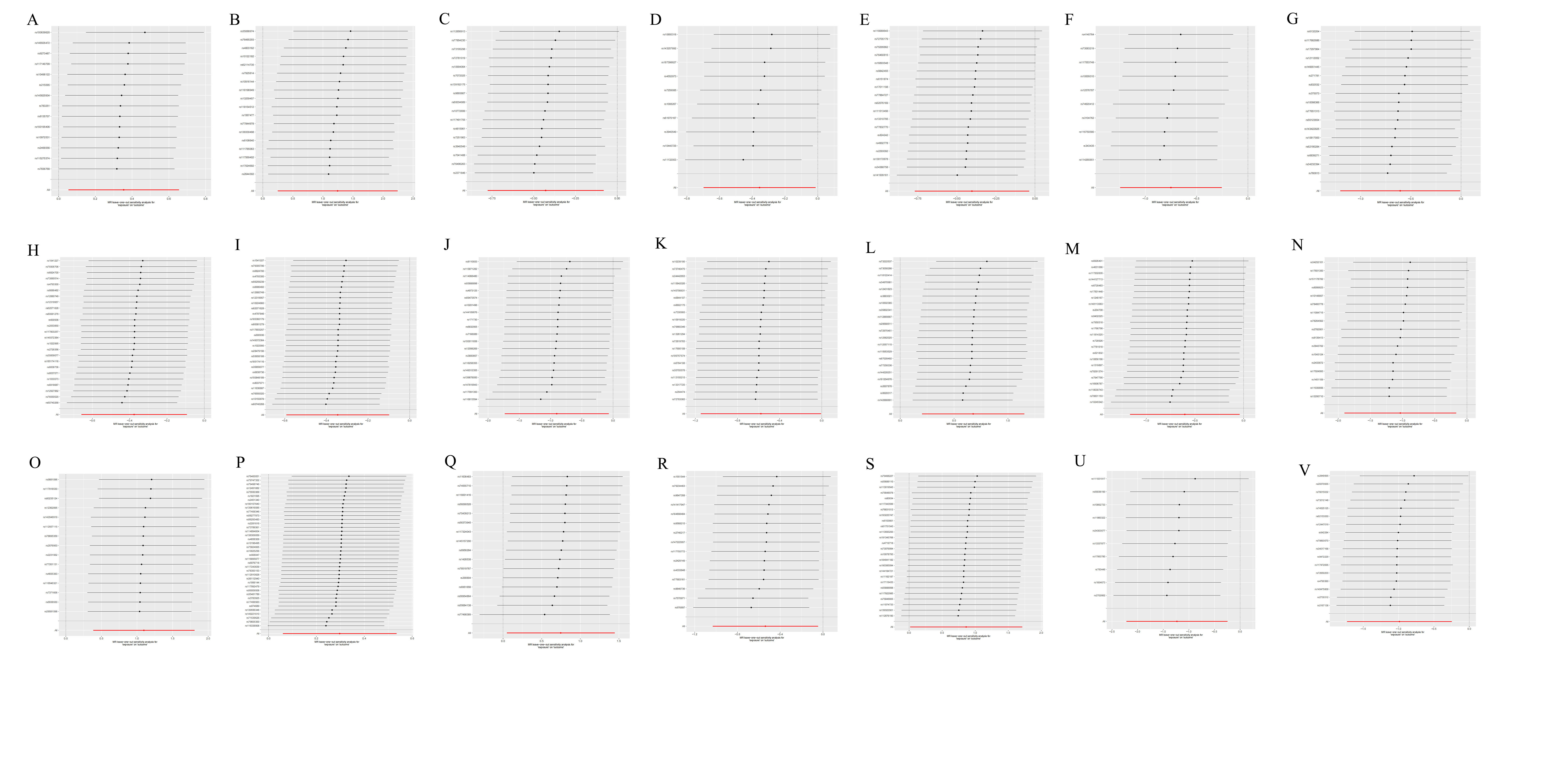

Supplement: Supplementary Figure 1 — Leave-one-out plot to visualize the causal effect of each GM associated with nonunion when leaving one SNP out. (A) Agathobacter sp000434275. (B) Aureimonas. (C) Bifidobacterium breve. (D) Bifidobacterium kashiwanohense. (E) Blautia hansenii. (F) CAG-145. (G) CAG-488 sp000434055. (H) CAG-877 sp000433455. (I) CAG-877. (J) Campylobacter D. (K). Clostridium E. (L) Clostridium. (M) Enterococcus A. (N) Kineothrix. (O) Lachnospirales. (P) Megamonas funiformis. (Q) Peptococcia. (R) Phocea massiliensis. (S) Phocea massiliensis. (T) Phocea massiliensis. (U) Rhodococcus. (V) UBA1777 sp900319835. [file Image_1.jpeg]

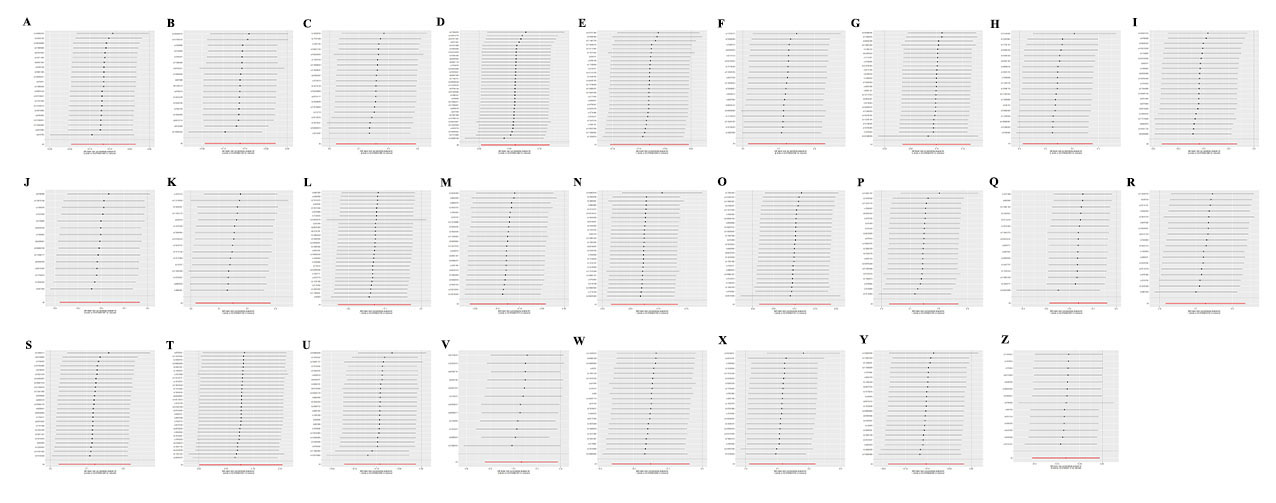

Supplement: Supplementary Figure 2 — Leave-one-out plot to visualize the causal effect of each immune cell associated with nonunion when leaving one SNP out. (A) IgD– CD38br AC. (B) PB/PC %lymphocyte. (C) CD86+ plasmacytoid DC AC. (D) Resting Treg %CD4. (E) CD39+ secreting Treg %secreting Treg. (F) EM CD4+ %T cell. (G) TD CD4+ %T cell. (H) T cell AC. (I) CD28– CD8br %CD8br. (J) CD28– CD8br AC. (K) CD19 on IgD– CD38br. (L) CD20 on memory B cell. (M) CD24 on IgD+ CD38– unsw mem. (N) CD25 on IgD+ CD38–. (O) CD25 on naive-mature B cell. (P) CD25 on transitional. (Q) CD27 on PB/PC. (R) CD38 on CD3– CD19–. (S) IgD on IgD+ CD24+. (T) CD3 on T cell. (U) CD28 on activated & secreting Treg. (V) CD28 on CD45RA– CD4 not Treg. (W) FSC-A on granulocyte. (X) FSC-A on T cell. (Y) CD4 on CD39+ secreting Treg. (Z) HLA DR on CD33– HLA DR+. [file Image_2.jpeg]
